# Management of an Oral Squamous Cell Papilloma in an Unusual Maxillofacial Location: A Case Report

**DOI:** 10.7759/cureus.101197

**Published:** 2026-01-09

**Authors:** Marimuthu P, Priti Shukla, Neha Saini, Keerthy Ajay Kumar, Niraj Prasad

**Affiliations:** 1 Oral and Maxillofacial Surgery, All India Institute of Medical Sciences, Raebareli, Raebareli, IND; 2 Orthodontics, All India Institute of Medical Sciences, Raebareli, Raebareli, IND; 3 Anatomy, All India Institute of Medical Sciences, Raebareli, Raebareli, IND; 4 Pathology and Laboratory Medicine, All India Institute of Medical Sciences, Raebareli, Raebareli, IND

**Keywords:** benign oral lesion, human papillomavirus, oral squamous cell papilloma, pterygomandibular region, surgical option

## Abstract

According to the World Health Organization (WHO) classification, oral squamous cell papilloma is a benign, hyperplastic, exophytic proliferation characterized by a verrucous or cauliflower-like morphology, with either a sessile or pedunculated base. It is among the most common benign epithelial lesions of the oral cavity, showing a predilection for the hard and soft palate. Although human papillomavirus (HPV) infection has been implicated in its pathogenesis, the precise viral role remains controversial.

We describe a rare case of oral squamous cell papilloma arising in the pterygomandibular region of the right mandible - an uncommon site for this lesion. The patient presented with a localized, asymptomatic, exophytic growth that had been slowly enlarging over time. Clinical examination revealed a well-defined, nontender lesion. Excisional biopsy was performed under local anesthesia, and histopathological analysis confirmed the diagnosis of squamous papilloma, showing finger-like projections of stratified squamous epithelium with fibrovascular cores. The postoperative period was uneventful, and no recurrence was noted during follow-up. Oral squamous cell papilloma is a benign lesion with distinct histopathological features and an excellent prognosis. While it commonly affects the palate, occurrence in the pterygomandibular region is extremely rare. Awareness of such atypical presentations is essential for accurate diagnosis and appropriate clinical management.

## Introduction

Squamous cell papilloma is a common benign epithelial lesion of the oral mucosa, most frequently affecting the tongue, the vermilion border of the lips, the uvula, and the mucosa of the hard and soft palate. Clinically, it presents as an exophytic growth, often raising concern due to its appearance, although it is a benign and noncontagious lesion [1]. The pathogenesis of oral squamous cell papilloma remains uncertain; however, many oral and maxillofacial surgeons and pathologists support its association with human papillomavirus (HPV) infection. This theory is based on its clinical resemblance to cutaneous warts and the detection of HPV subtypes 2, 6, 11, and 57 in certain cases of oral squamous cell papilloma [2]. Regardless of its etiology, squamous cell papilloma typically arises in common intraoral sites but can occur in any region of the oral mucosa. It usually appears as a solitary, asymptomatic lesion without induration, exhibiting either a sessile base or, less commonly, a pedunculated stalk.

This report presents a rare presentation of oral squamous cell papilloma in the pterygomandibular region and highlights key diagnostic and management considerations. In the pterygomandibular region, lesions that occur are usually oral carcinoma. Hence, it is clinically important for surgeons to be aware of this unusual location of squamous cell papilloma.

## Case presentation

**Figure 1:**
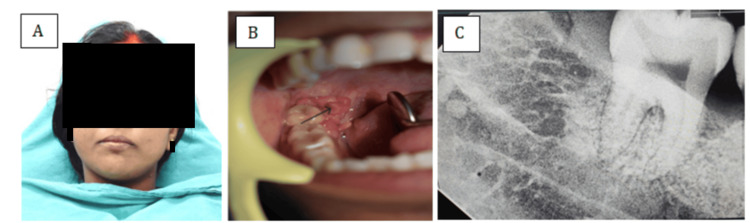
A) Extraoral image of the patient; B) Intraoral image of the patient (the arrow indicates the intraoral lesion); C) Preoperative intraoral periapical radiograph

A 33-year-old female patient reported to the Department of Dentistry, All India Institute of Medical Sciences (AIIMS), Raebareli, with the chief complaint of a growth in the right lower posterior region of the oral cavity for the past eight months. The lesion was initially small and had gradually increased in size over the past eight months. It was asymptomatic, with no history of pain, pus discharge, or bleeding. On extra oral examination, reveals no significant abnormalities (Figure 1A).

On intraoral examination, a single exophytic, sessile growth was observed in the pterygomandibular region, extending onto the lingual surface of the mandibular second and third molars. The lesion measured approximately 4 × 3 cm, was pink in color, had a pebbled surface, and was soft in consistency, with no tenderness on palpation and no ulceration or bleeding. No similar growths were noted elsewhere in the oral cavity or on the extremities (Figure 1B). There was no evidence of regional lymphadenopathy.

The patient’s medical and surgical histories were non-contributory. There was no history of tobacco use, immunosuppression, oral habits, or HPV-related conditions. Routine hematological investigations were within normal limits. Preoperative intraoral periapical radiograph revealed no underlying bony involvement or radiographic abnormalities (Figure 1C). Based on the clinical presentation, a provisional diagnosis of squamous papilloma was considered, and the lesion was planned for surgical excision under local anesthesia for histopathological confirmation.

**Figure 2:**
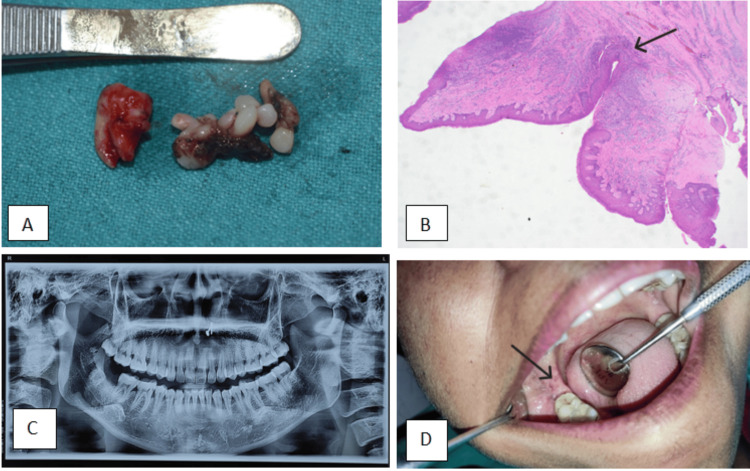
A) Completely excised lesion along with tooth; B) Histopathology image (H&E ×10) of the specimen (the arrow indicates prominent fibrovascular cores); C) Postoperative panoramic radiograph; D) Postoperative image of the surgical site (the arrow indicates postoperative healing of surgical site)

An excisional biopsy of the lesion was performed under local anesthesia. Complete surgical excision of the lesion was achieved, including a small margin of normal surrounding tissue to minimize the risk of recurrence and promote optimal healing. Wound closure was performed using non-absorbable 3-0 silk sutures, and the excised specimen was submitted for histopathological examination (Figure 2A). Postoperative instructions were provided, and follow-up visits were scheduled to monitor healing.

Histopathological examination revealed papillary proliferations of stratified squamous epithelium with parakeratosis and focal ulceration, covered by surface exudate. The epithelial cells maintained normal polarity, and the papillary projections exhibited prominent fibrovascular cores. The underlying connective tissue stroma demonstrated dense lymphoplasmacytic inflammatory infiltrate and focal areas of hyalinization. These microscopic features confirmed the diagnosis of squamous papilloma (Figure 2B).

The postoperative course was uneventful, and follow-up examinations at one week, three months, and six months revealed satisfactory healing, with a panoramic radiograph showing no changes (Figures 2C-2D).

## Discussion

The term oral squamous cell papilloma refers to a verrucous, papillary, or sometimes cauliflower-like growth composed of benign stratified squamous epithelium supported by fibrovascular connective tissue. It is commonly associated with HPV types 6 and 11 [3]. Oral squamous papilloma represents the most frequent papillary lesion of the oral mucosa, including the vermilion border of the lips and accounts for approximately 2.5% of all oral lesions.

In the present case, the lesion occurred in the pterygomandibular region - an uncommon location for squamous papilloma. The etiological relationship between intraoral squamous papillomas and cutaneous verruca vulgaris (warts) remains unclear. The lesion is characterized by low virulence and is noncontagious. Some authors have proposed that oral squamous papilloma may represent a reactive epithelial proliferation secondary to minor trauma, rather than a true neoplastic process [4].

Recent studies have demonstrated a close association between HPV infection and the development of squamous papilloma [5]. In adults, HPV transmission is typically sexual, whereas in children, infection may occur through vertical transmission during childbirth or via contact with infected epithelial cells.

Oral squamous cell papilloma is a benign epithelial lesion with characteristic clinical and histopathological features. Although it most frequently affects the tongue, lips, and palate, its occurrence in the pterygomandibular region is extremely rare. Awareness of such atypical presentations is important for clinicians to include this lesion in the differential diagnosis of exophytic oral growths. Complete surgical excision remains the treatment of choice, providing an excellent prognosis with minimal risk of recurrence. Early recognition and appropriate management ensure favorable outcomes and reduce patient concern regarding the lesion’s appearance or potential malignancy [6,7].

## Conclusions

Oral squamous papilloma is a benign epithelial proliferation, often associated with HPV and most often located on the palate or tongue. The present case is notable due to its rare occurrence in the pterygomandibular region, a site infrequently documented in the literature. Clinical examination and histopathology are key; imaging can help assess underlying bone involvement when indicated. Excision is the standard treatment; recurrence is generally uncommon, and follow-up is recommended. Increased awareness of such atypical presentations enhances diagnostic accuracy and supports more effective clinical decision-making. However, a limitation of our study was that no HPV testing was performed. Therefore, future follow-up can include HPV testing to better understand prognosis over a longer period.
